# Moderation Effects of Streetscape Perceptions on the Associations Between Accessibility, Land Use Mix, and Bike-Sharing Use: Cross-Sectional Study

**DOI:** 10.2196/58761

**Published:** 2024-07-03

**Authors:** Huagui Guo, Shuyu Zhang, Xinwei Xie, Jiang Liu, Hung Chak Ho

**Affiliations:** 1 School of Architecture and Urban-Rural Planning, Fuzhou University, Fuzhou, China; 2 Laboratory of Smart Habitat for Humanity, Fuzhou University, Fuzhou, China; 3 Department of Public and International Affairs, City University of Hong Kong, Hong Kong, China

**Keywords:** built environment, streetscape perceptions, bike-sharing use, cycling, moderation effect, China

## Abstract

**Background:**

Cycling is known to be beneficial for human health. Studies have suggested significant associations of physical activity with macroscale built environments and streetscapes. However, whether good streetscapes can amplify the benefits of a favorable built environment on physical activity remains unknown.

**Objective:**

This study examines whether streetscape perceptions can modify the associations between accessibility, land use mix, and bike-sharing use.

**Methods:**

This cross-sectional study used data from 18,019,266 bike-sharing orders during weekends in Shanghai, China. A 500 × 500 m grid was selected as the analysis unit to allocate data. Bike-sharing use was defined as the number of bike-sharing origins. Street view images and a human-machine adversarial scoring framework were combined to evaluate lively, safety, and wealthy perceptions. Negative binomial regression was developed to examine the independent effects of the three perceptual factors in both the univariate model and fully adjusted model, controlling for population density, average building height, distance to nearest transit, number of bus stations, number of points of interest, distance to the nearest park, and distance to the central business district. The moderation effect was then investigated through the interaction term between streetscape perception and accessibility and land use mix, based on the fully adjusted model. We also tested whether the findings of streetscape moderation effects are robust when examinations are performed at different geographic scales, using a small-sample statistics approach and different operationalizations of land use mix and accessibility.

**Results:**

High levels of lively, safety, and wealthy perceptions were correlated with more bike-sharing activities. There were negative effects for the interactions between the land use Herfindahl-Hirschman index with the lively perception (β=–0.63; *P*=.01) and safety perception (β=–0.52; *P*=.001). The interaction between the lively perception and road intersection density was positively associated with the number of bike-sharing uses (β=0.43; *P*=.08). Among these, the lively perception showed the greatest independent effect (β=1.29; *P*<.001), followed by the safety perception (β=1.22; *P*=.001) and wealthy perception (β=0.72; *P*=.001). The findings were robust in the three sensitivity analyses.

**Conclusions:**

A safer and livelier streetscape can enhance the benefits of land use mix in promoting bike-sharing use, with a safer streetscape also intensifying the effect of accessibility. Interventions focused on streetscape perceptions can encourage cycling behavior and enhance the benefits of accessibility and land use mix. This study also contributes to the literature on potential moderators of built environment healthy behavior associations from the perspective of microscale environmental perceptions.

## Introduction

Several studies have demonstrated that a favorable built environment can promote walking and bike-sharing use, particularly in Chinese cities [1-3]. However, findings on the effects of built environments are usually mixed [4-6]. One potential explanation is that there may be interactions between built environments at different levels, including the moderation effects of streetscape perceptions [7,8]. An in-depth understanding of streetscape perceptions as moderators is not only an important methodological issue for environmental health research [9] but also informative for creating effective interventions in built environments. Despite some efforts [10,11], however, it remains unclear whether and to what extent good perceptions of streetscapes can enhance the benefits of favorable built environments.

The ecological model of physical activity provides a theoretical foundation for streetscape moderation effects. According to this model, one’s cycling behavior is determined by factors across multiple levels [9]. Such behavior is shaped by interactions among indicators at different levels [9]. Moreover, understanding the benefits of streetscapes in promoting health is important. Microscale streetscapes provide numerous benefits to people’s physical activity [11-13]. Additionally, the built environment at the neighborhood level has essentially been established and fixed over time, and streets have become a focus of high-quality urban development in the era of inventory planning, particularly in Chinese cities.

However, little attention has been given to the moderation effects of streetscapes [8,14,15]. Furthermore, most studies examining environmental perception as a moderator focus on perceived safety [16-18], while little attention has been given to other perceived aspects. Moreover, streetscapes as potential moderators are usually examined from the objective side [8,10] and are less understood from a perceptual perspective. It has been reported that residents’ safety perceptions are more likely to be correlated with physical activity [8,19], especially for those who do not perceive threats from neighborhoods with high crime rates. However, insufficient attention has been given to streetscapes in studies exploring environmental moderation from a perception perspective [8].

To fill the aforementioned gaps, this study aims to address whether streetscape perceptions can modify the effects of accessibility and land use mix on bike-sharing use. To achieve this, we used data from 18,019,266 GPS-based bike-sharing orders and conducted a cross-sectional study in Shanghai, China. This study contributes to the literature on the potential moderation effects of urban environments from the perspective of microscale environmental perception [8,18,20]. The findings are derived from Chinese cities, where urban forms and the leading perception factors as moderators differ significantly from those of Western countries [10,21], which all advance the development of ecological models of health behavior [9]. This cross-sectional study, with its large data volume and spatial coverage, suggests that urban planning initiatives aiming to encourage healthy behaviors among residents should consider microscale streetscapes, which can promote physical activities and enhance the benefits of accessibility and land use mix.

## Methods

### Research Area

**Figure 1.**
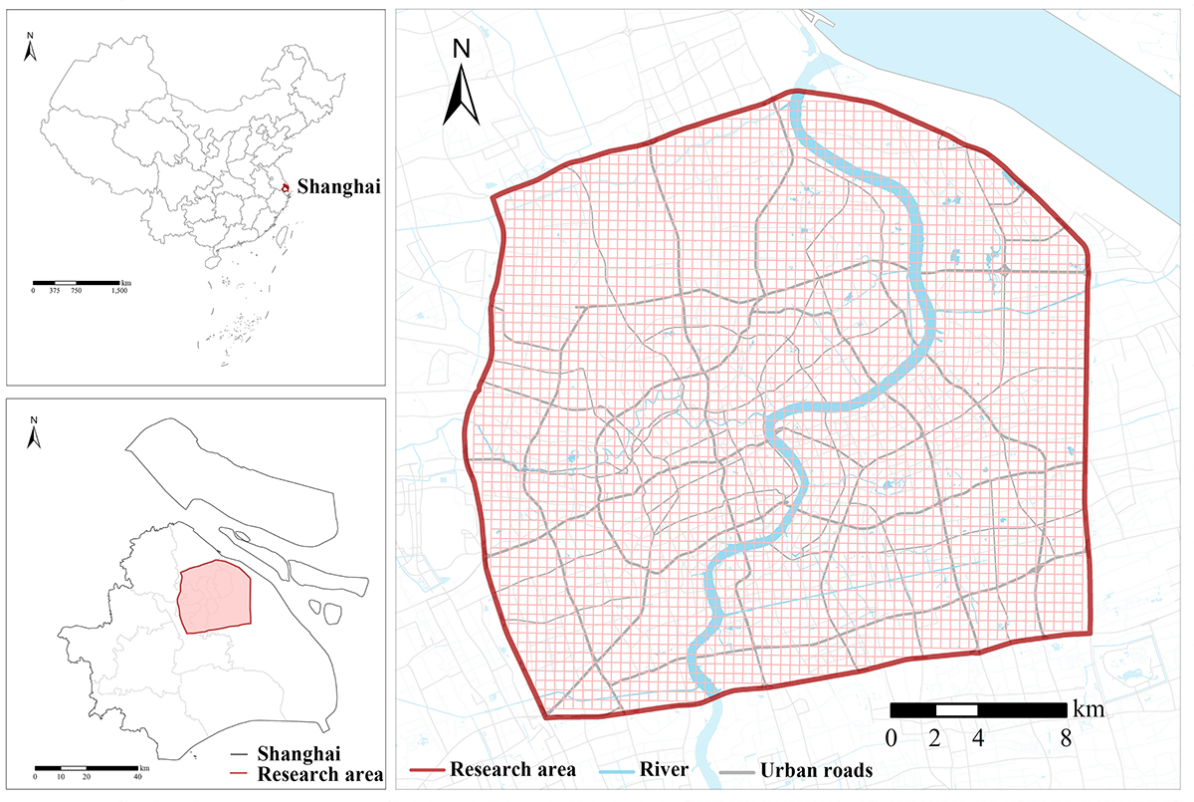
Research area delineated by the Outer Ring Highway of Shanghai, China.

We examined the moderation effects of streetscape perceptions in central Shanghai, China. The research area was delineated by the Outer Ring Highway of Shanghai (Figure 1), mainly because of the availability of street view images and bike-sharing use data. As one of the four first-tier cities in China, Shanghai is among the largest cities in the world. As of 2017, there were 1.5 million dockless bikes in Shanghai. The terrain is flat, and the climate is relatively favorable, which provides conducive conditions for people’s cycling activities.

### Data Source and Research Design

This is a cross-sectional study in nature, with grids as analysis units to allocate data. Bike-sharing use data were provided by the Mobike Technology Co., Ltd, spanning from August 26 to September 8, 2018. Originally, the number of bike-sharing use records was about 19 billion. Each record included information on trip ID, start time and location, and end time and location. As in many studies [2,10], records on rainy days (eg, September 6, 2018) were not included. Additionally, we excluded records with abnormal durations or lengths, retaining records with a trip duration from 2 minutes to 1 hour. After excluding trips with locations out of our research area, the final number of bike-sharing use records in this study was 18,019,266.

Data on street view images were obtained from the Baidu application programming interface [22], with the sampling points at 50-m intervals along the road network. Each sampling point contains street view images with four headings. Data used to measure macroscale built environments included population data derived from EasyGO data and road networks, buildings, and points of interest (POIs) acquired from Baidu Maps [22].

### Deriving Bike-Sharing Use

**Figure 2.**
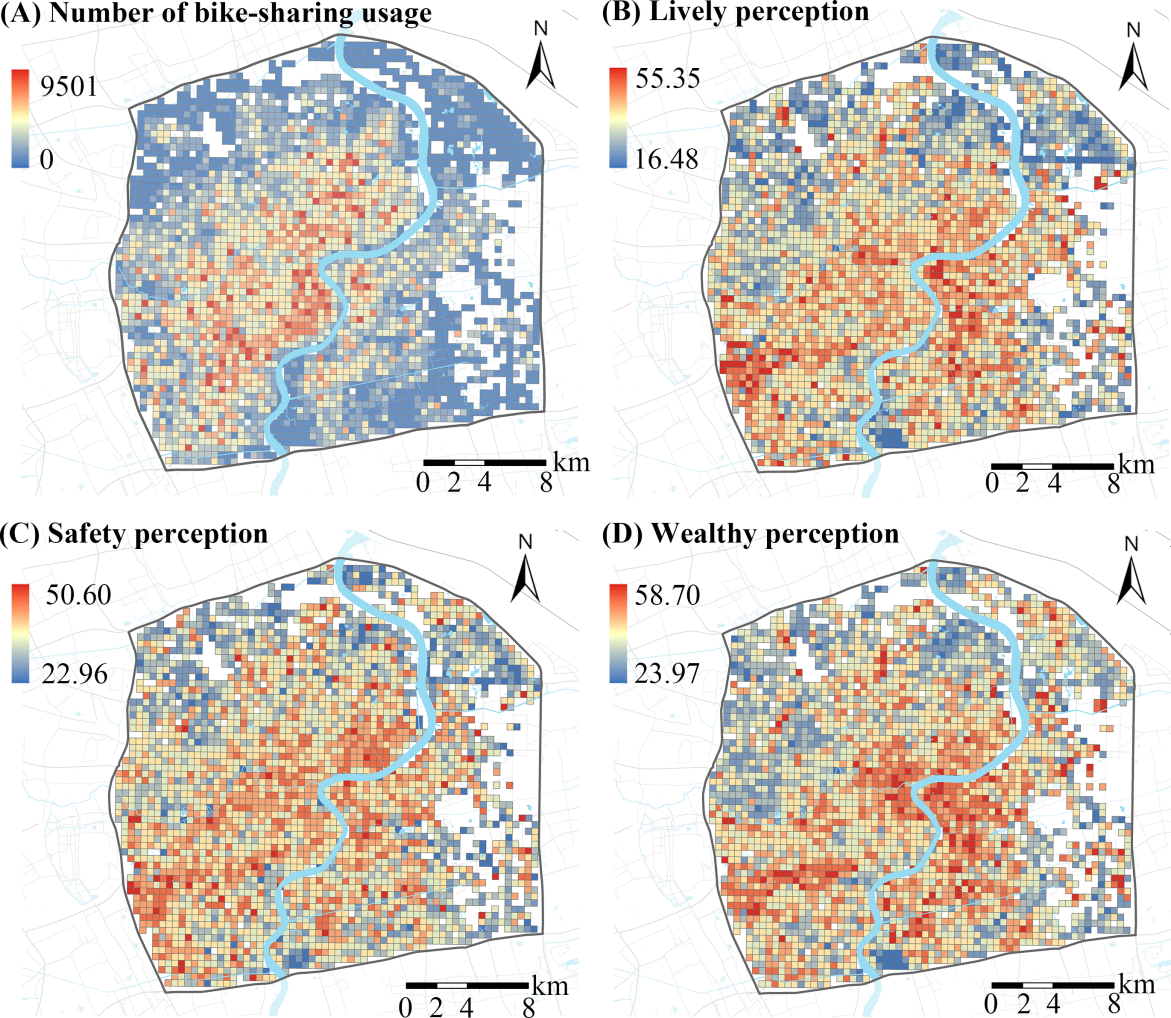
Spatial distributions of the number of bike-sharing uses and assessed streetscape perceptions during 2018 in Shanghai, China.

Bike-sharing use is defined as the number of bike-sharing origins on weekends in each grid. Similar to studies examining environmental effects on bike-sharing use [10,23], a 500 × 500 m grid was selected as the analysis unit to allocate each trip, primarily because the size of the grids selected was usually considered to be the main activity space in Chinese neighborhoods [21,24]. Table S1 in Multimedia Appendix 1 presents the summary statistics of bike-sharing use, streetscape perceptions, and macroscale built environments. Figure 2A exhibits the spatial distributions of bike-sharing origins on weekends.

### Assessing Streetscape Perceptions

Streetscape perceptions are three positive perceptual indicators (ie, lively, safety, and wealthy) [25]. They were assessed according to a human-machine adversarial scoring framework developed by Yao et al [25]. Briefly, volunteers scored the perceptions based on the displayed street view image, with the scored values ranging from 0 to 100. Subsequently, a random forest model was used to fit the association of volunteer scorings with visual scenic features extracted by the segmentation approach of the fully convolutional neural network–8. Once the first 50 photos were scored by a volunteer, a random forest set was created to fit the perceived scores in the scoring software. When the volunteer rated the subsequent photos, the software provided the recommended scores based on the rules learned from the volunteer’s previous rating. In this process, an iterative feedback module was used to automatically adjust the recommended scores according to the user’s scoring behaviors. When the difference between the recommended and scored values was lower than 5 points, the scoring procedure stopped and a human-machine adversarial scoring module was created.

**Figure 3.**
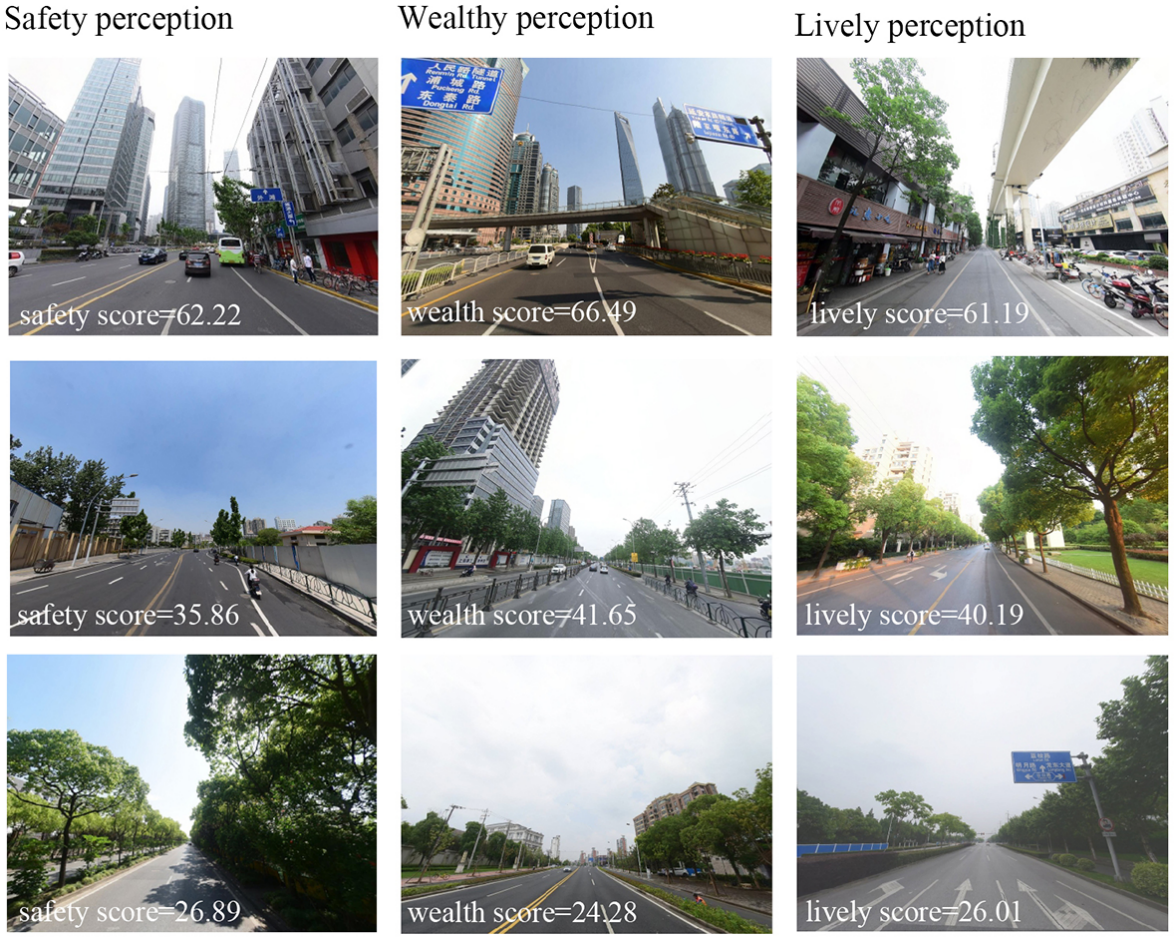
Examples of the assessment for lively, safety, and wealthy perceptions during 2018 in Shanghai, China.

As reported, errors in the estimated perceptions using the scoring framework were less than 10% [25]. In this situation, the created scoring module was used to assess the three perceptual indicators and then obtain the perceptual scores for each street view image. The perceptual scores for each sampling point were then calculated by averaging the perceptual scores of each of the four images. Finally, perception scores for each grid were calculated by averaging the perceptual scores of sampling points located in the grid. Figures 2 (B-D) and 3 show the spatial distributions of the three perception indicators assessed and their examples.

### Measuring Macroscale Built Environments

**Figure 4.**
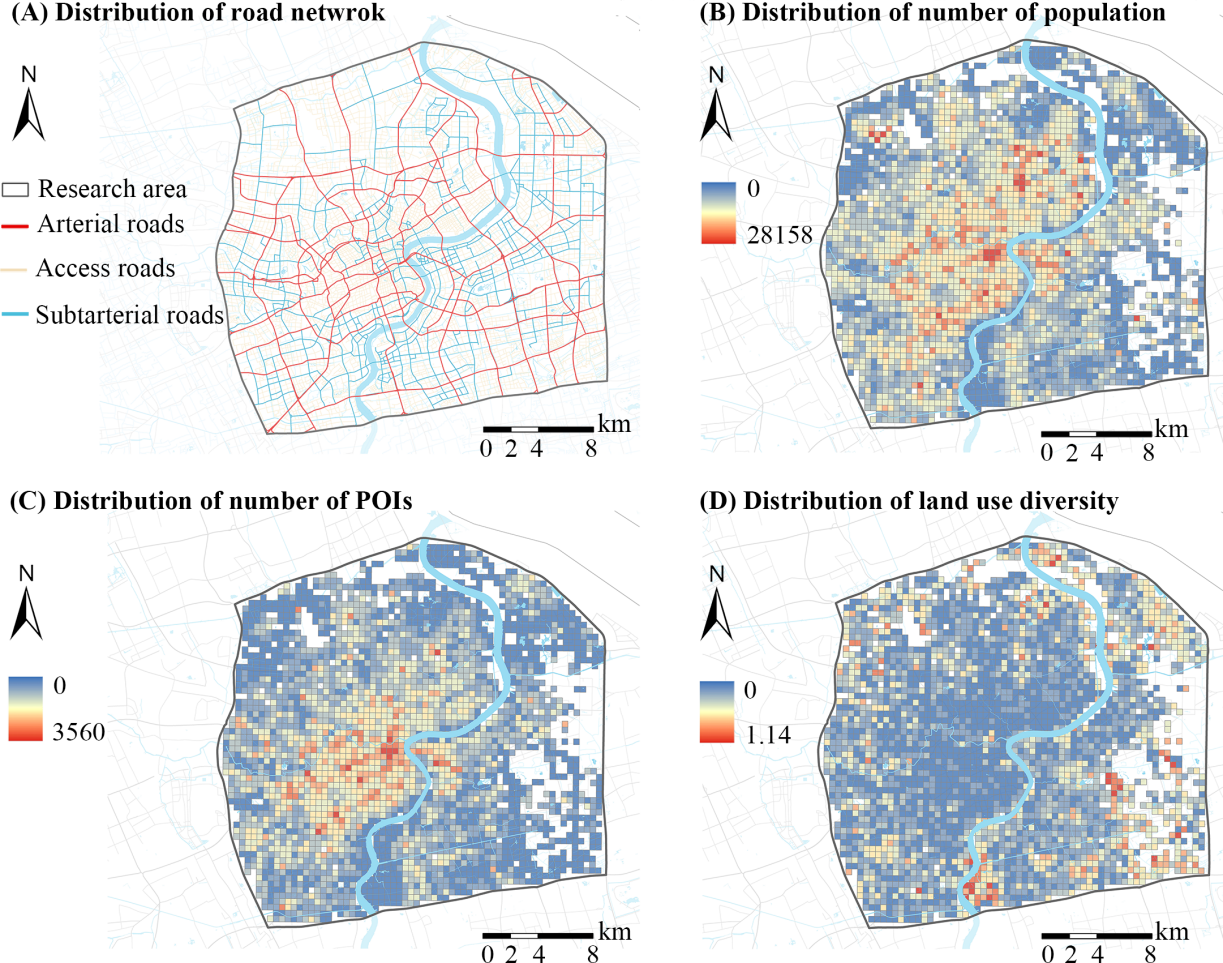
Spatial distributions of some built environment elements in 2018 at macroscale in Shanghai, China. POI: point of interest.

Macroscale built environments were measured in terms of the 5Ds framework [1]. Population and average building height were used to measure density. The land use Herfindahl-Hirschman index (HHI) was used to assess diversity, with a high value indicating a less mixed degree of different types of land use [26]. Destination accessibility was measured using the number of POIs and distance to the nearest park. Distance to transit was assessed in terms of distance to the nearest metro station and the number of bus stops. The design was measured through the number of road intersections. Distance to the central business district (CBD) was operationalized as the distance between the centroid of a grid and the CBD (km) to control for the effect of location. Figure 4 shows the spatial distributions of macroscale built environment factors.

### Statistical Analysis

The negative binomial regression model (NBRM) was used to examine the independent effects of streetscape perceptions. This was performed in the univariate model and then the fully adjusted model, controlling for population density, average building height, distance to nearest transit, number of bus stations, number of POIs, distance to the nearest park, and distance to CBD. The NBRM was selected primarily because the outcome variable (ie, the number of bike-sharing origins) was in number format and discrete. Moreover, the variance of the outcome variable is greater than its mean value. Hence, as in many studies [27,28], the NBRM was chosen for the examination.

Subsequently, streetscape moderation effects were examined through the interaction term between macroscale built environment factors and streetscape perceptions using the fully adjusted NBRM. Factors at the macroscale level included land use mix and accessibility, while the three perception indicators were lively, safety, and wealthy. Consequently, 6 total models were produced, with each model examining different combinations of these factors. To mitigate multicollinearity and facilitate comparison between independent variables, independent factors were all standardized into *z* scores.

Finally, we conducted three sensitivity analyses. Recognizing the potential influence of an uncertain geographic context problem [29], we examined whether the results of streetscape moderation roles are sensitive when investigations are performed at different geographic scales. Consistent with previous studies [3,30], a 1000 × 1000 m grid was selected as the analysis unit. Furthermore, apart from the big data analysis, we further tested whether there were moderation effects of streetscapes using a classic (small sample) statistics approach. Similar to the sampling rate of prior studies [10,31], 20% of the total grids were randomly chosen for further examination. Moreover, we tested streetscape moderation roles when land use mix and accessibility were operationalized differently. Road length and land use HHI calculated based on area of interest data were used as further proxies.

### Ethical Considerations

This study was approved by the Human and Artefacts Ethics Sub-Committee at the City University of Hong Kong (reference 27609824). The bike-sharing record data were anonymous and deidentified, including information on trip ID, start time and location, and end time and location, in this study.

## Results

### Effects of Streetscape Perceptions

In the univariate model, each of the three positive perceptual factors was positively associated with the number of bike-sharing uses on weekends (Table S2 in Multimedia Appendix 1). Among this, the lively perception showed the greatest effect (β=5.50; *P*<.001), followed by the safety perception (β=5.38; *P*<.001) and wealthy perception (β=4.45; *P*<.001). A similar pattern of results was observed in the fully controlled model (Table 1). In particular, the most influential effect was found for the lively perception (β=1.45; *P*<.001), followed by the safety perception (β=1.19; *P*<.001) and wealthy perception (β=0.79; *P*<.001).

**Table 1.** Effects of lively, safety, and wealthy perceptions on the number of bike-sharing uses on weekends in 2018 in Shanghai, China.

Independent variables	For lively		For safety		For wealthy	
	β (SD)	*P* values	β (SD)	*P* values	β (SD)	*P* values
Perception	0.16 (0.02)	<.001	0.12 (0.02)	<.001	0.08 (0.02)	<.001
Population	0.58 (0.03)	<.001	0.57 (0.03)	<.001	0.59 (0.03)	<.001
Average building height	0.08 (0.01)	<.001	0.09 (0.01)	<.001	0.09 (0.01)	<.001
Land use mix	–0.21 (0.02)	<.001	–0.22 (0.02)	<.001	–0.22 (0.02)	<.001
Number of road intersections	0.05 (0.02)	.002	0.05 (0.02)	<.001	0.05 (0.02)	<.001
Distance to nearest transit	–0.30 (0.03)	<.001	–0.30 (0.02)	<.001	–0.30 (0.03)	<.001
Number of bus stations	0.12 (0.01)	<.001	0.13 (0.01)	<.001	0.12 (0.01)	<.001
Number of POIs^a^	0.11 (0.02)	<.001	0.11 (0.02)	<.001	0.12 (0.02)	<.001
Distance to nearest parks	–0.21 (0.02)	<.001	–0.20 (0.02)	<.001	–0.22 (0.02)	<.001
Distance to CBD^b^	–0.17 (0.03)	<.001	–0.17 (0.03)	<.001	–0.17 (0.03)	<.001

a POI: point of interest.

b CBD: central business district.

### Moderation Effects of Streetscape Perceptions

All streetscape perceptions positively moderated the association between the land use HHI and the number of bike-sharing uses on weekends (Table 2). Specifically, streetscape perceptions and the land use HHI were significantly associated with the number of bike-sharing uses on weekends; among the three perceptual indicators, the lively perception showed the greatest effect. Regarding the moderation effect, the effect of the interaction between the land use HHI and each of the streetscape perceptions was smaller than that of each of the two factors; the lively perception presented the strongest moderation role on the effect of the land use HHI, followed by the wealthy perception and safety perception. Specifically, the effect of the interaction between the lively perception and the land use HHI was –0.63 (*P*=.01), greater than those of interactions between the land use HHI with the wealthy and safety perceptions at –0.60 (*P=*.008) and –0.52 (*P*=.001), respectively. This means that livelier, safer, and wealthier street environments can enhance the benefits of land use mix to promote cycling behavior.

There were positive interactions in the number of road intersections with the lively and wealthy perceptions (Table 3). The effects of the number of road intersections, both of the two perceptions, and their interactions were all positive. Different from that of land use mix, the effect of the number of road intersections was close to the impact of the interaction with the lively and wealthy perceptions. With respect to the moderation role, the lively perception showed the largest moderation effect, although no significant moderation effect was observed for the safety perception. This means that good lively and wealthy perceptions of streetscapes are likely to magnify the role of accessibility in encouraging cycling activities.

**Table 2.** Moderation effects of lively, safety, and wealthy perceptions on the association between land use mix and bike-sharing use on weekends in 2018 in Shanghai.

Independent variables	For lively		For safety		For wealthy	
	β (SD)	*P* values	β (SD)	*P* values	β (SD)	*P* values
Perception	0.13 (0.02)	<.001	0.12 (0.02)	<.001	0.07 (0.02)	<.001
Population	0.57 (0.03)	<.001	0.57 (0.03)	<.001	0.58 (0.03)	<.001
Average building height	0.08 (0.01)	<.001	0.09 (0.01)	<.001	0.08 (0.01)	<.001
Land use mix	–0.25 (0.03)	<.001	–0.25 (0.02)	<.001	–0.25 (0.02)	<.001
Number of road intersections	0.04 (0.02)	.01	0.05 (0.02)	<.001	0.05 (0.02)	<.001
Distance to nearest transit	–0.29 (0.03)	<.001	–0.30 (0.02)	<.001	–0.30 (0.03)	<.001
Number of bus stations	0.12 (0.01)	<.001	0.12 (0.01)	<.001	0.12 (0.01)	<.001
Number of POIs^a^	0.12 (0.02)	<.001	0.11 (0.02)	<.001	0.13 (0.02)	<.001
Distance to nearest parks	–0.16 (0.03)	<.001	–0.17 (0.03)	<.001	–0.17 (0.03)	<.001
Distance to CBD^b^	–0.24 (0.03)	<.001	–0.23 (0.02)	<.001	–0.23 (0.02)	<.001
Land use mix × perception^c^	–0.63 (0.03)	.01	–0.52 (0.02)	<.001	–0.60 (0.02)	.01

a POI: point of interest.

b CBD: central business district.

c For value = original value × 10.

**Table 3.** Moderation effects of lively, safety, and wealthy perceptions on the associations between accessibility and bike-sharing use on weekends in 2018 in Shanghai, China.

Independent variables	For lively		For safety		For wealthy	
	β (SD)	*P* values	β (SD)	*P* values	β (SD)	*P* values
Perception	0.14 (0.02)	<.001	0.12 (0.02)	<.001	0.08 (0.02)	<.001
Population	0.57 (0.03)	<.001	0.57 (0.03)	<.001	0.57 (0.03)	<.001
Average building height	0.08 (0.01)	<.001	0.09 (0.01)	<.001	0.08 (0.01)	<.001
Land use mix	–0.21 (0.02)	<.001	–0.22 (0.02)	<.001	–0.22 (0.02)	<.001
Number of road intersections	0.05 (0.01)	.001	0.05 (0.01)	<.001	0.06 (0.02)	<.001
Distance to nearest transit	–0.29 (0.03)	<.001	–0.30 (0.02)	<.001	–0.30 (0.03)	<.001
Number of bus stations	0.18 (0.01)	<.001	0.12 (0.01)	<.001	0.12 (0.01)	<.001
Number of POIs^a^	0.11 (0.02)	<.001	0.11 (0.02)	<.001	0.13 (0.02)	<.001
Distance to nearest park	–0.17 (0.03)	<.001	–0.17 (0.03)	<.001	–0.17 (0.03)	<.001
Distance to CBD^b^	–0.26 (0.03)	<.001	–0.21 (0.02)	<.001	–0.21 (0.02)	<.001
Number of road intersections × perception^c^	0.43 (0.02)	.08	0.17 (0.02)	.29	0.39 (0.02)	<.001

a POI: point of interest.

b CBD: central business district.

c For value = original value × 10.

### Sensitivity Analyses

**Figure 5.**
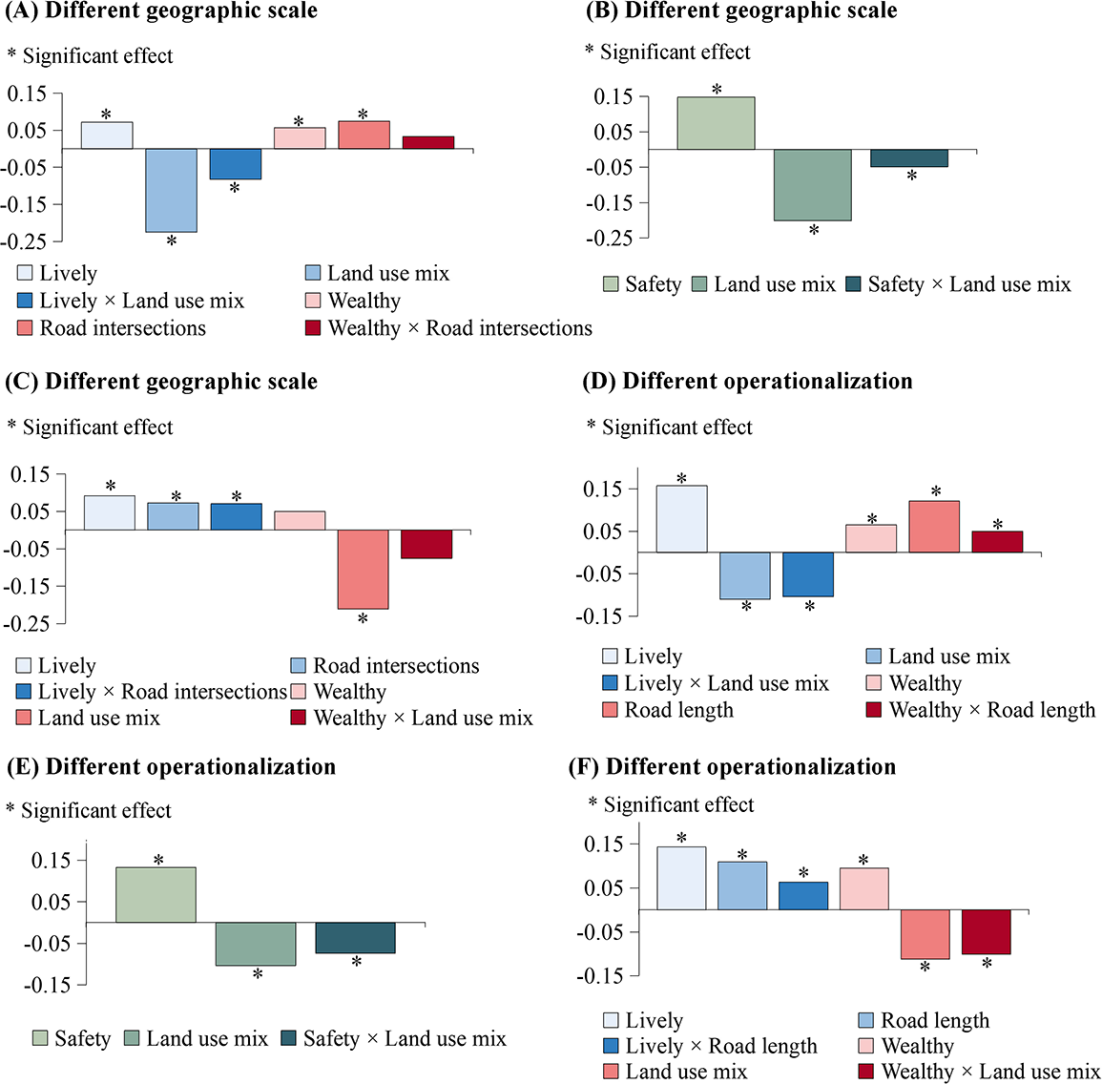
Sensitivity analyses of streetscape moderation effects examined at different geographic scales (A-C) and using different operationalizations (D-F) in 2018 in Shanghai, China.

As indicated in the sensitivity analysis using a 1000 × 1000 m grid as the analysis unit (Figure 5 A-C), there were significant interactions between land use mix (ie, land use HHI) and both lively and safety perceptions of the streetscape, but not the wealthy perception. Similarly, a moderation role on the impact of the number of road intersections was found for the lively perception but not for the wealthy perspective. Figure S1 G-I in Multimedia Appendix 1 illustrates the sensitivity analysis in terms of a randomly selected small sample. Overall, positive moderation roles persisted for all streetscape perceptions on the effect of land use mix and for the lively perception on the impact of the number of road intersections. Furthermore, among the three perception indicators, the lively perception still played the most substantial moderation role in relation to the impact of the land use HHI.

Figure 5 D-F exhibits the sensitivity analysis using the land use HHI calculated using area of interest data and road length as proxies for land use mix and accessibility, respectively. Generally, lively, wealthy, and safety perceptions of streetscapes continued to exhibit positive moderation on the effect of the land use HHI. In particular, the absolute effect of the interaction between land use mix and lively perception was 0.10 (*P*=.001), accounting for 66.14% and 94.22% of the main effects of the two factors, respectively. With regards to accessibility, operationalized by road length in grids, its interactions with lively and wealthy perceptions were still correlated with the number of bike-sharing uses on weekends.

## Discussion

### Principal Findings and Comparisons With Prior Work

We found that streetscape perceptions were positively associated with bike-sharing activities. This is consistent with many prior studies [17,32,33]. In particular, a cross-sectional study conducted in Boston, Massachusetts using running data from the Strava Heatmap suggested the positive effects of wealthy and safety perceptions on the amount of running [13]. The mechanisms linking environmental perceptions and physical activity are highly complex and beyond the scope of this work, highlighting the need for further investigation, especially with evidence from a neuroscience perspective.

However, we observed that among three perceptual factors, the lively perception not only plays the strongest effect on bike-sharing use but also emerges as the most influential moderator, which is inconsistent with findings from Western studies [7,13,20]. Typically, among environmental perceptions such as safety and lively, the former usually plays the strongest moderation role on the impact of macroscale built environments in studies outside of mainland China [8,16,20]. In particular, a US study indicated that compared to perceived pleasure, the safety perception plays a greater moderation role in the impact of accessibility on healthy behaviors [8].

The underlying mechanisms behind the lively perception not only playing the largest independent effect but also showing the strongest moderation effect are highly complex. There are two potential explanations. On the one hand, among the environmental perceptions (qualities), safety plays a crucial role in physical activity in many Western cities [7,13,20]. However, the lively perception usually takes precedence in residents’ concerns about healthy behavior in the Chinese context [17,34]. This is also consistent with the findings of this study. Regarding accessibility, the main effect of the lively perception on the number of bike-sharing uses on weekends was 1.39 (*P*<.001), higher than that of the safety and wealthy perceptions at 1.12 (*P*<.001) and 0.75 (*P*<.001), respectively. A similar pattern of results was observed for land use mix, with the greatest main effect for the lively perception (β=1.39; *P*<.001), followed by the safety perception (β=1.22; *P*<.001) and wealthy perception (β=0.75; *P*<.001).

On the other hand, livelier places tend to provide various benefits beyond urban liveliness itself. In many Chinese cities, more vibrant and lively urban places are associated with large population inflow and increased greenery [35,36]. A large number of people on the street and more green space can strengthen the sense of safety [37-39], thus promoting healthy behaviors more effectively [8,10]. The findings extend the results of prior studies by highlighting that, in the Chinese context, a lively perception, rather than a safety perception, usually plays the strongest moderation effect on the built environment–healthy behavior relationship.

This study provides significant implications for public health. Usually, urban planning initiatives aiming to promote residents’ physical activities target interventions in built environments at the macroscale level, which are time-consuming and less actionable. Our findings suggest that favorable microscale environments can promote cycling behavior and enhance the benefits of accessibility and land use mix as well. This underscores the need for interventions on microscale streetscapes, which are less time-consuming and more readily modifiable than those on macroscale built environments.

### Limitations

Several limitations should be clarified. First, similar to many studies [6,8,19], the use of 500 × 500 m grids as the analysis unit to examine moderation effects of streetscape perceptions may be susceptible to the uncertain geographic context problem [29]. In our study, the selected analysis unit was usually considered as the main activity space in most Chinese neighborhoods [21,24]. Moreover, a 1000 × 1000 m grid was used as the analysis unit to test the robustness of streetscape moderation effects. However, careful research design is essential to address the uncertain geographic context problem in future work.

Second, this is an aggregated study in nature with grids to allocate bike-sharing data. Hence, as in many bike-sharing studies [2,19], the impacts of individual characteristics on cycling were not controlled. Furthermore, streetscape perceptions were assessed by the public in terms of street view images, which may collectively influence the findings. Despite the negligible differences between respondents’ socioeconomic characteristics reported in some prior studies [40,41], future research should associate environmental perceptions with riders to draw more scientifically grounded conclusions. Finally, further study can delve into the mechanisms of moderation roles by microscale urban environments, either on the objective or perceived side, which remain unclear and could be better understood with individual-level data.

### Conclusions

Livelier and safer perceptions of streetscapes can magnify the benefits of land use mix for cycling activity; the lively perception has a similar effect on accessibility. The findings emphasize that sufficient microscale streetscape interventions can encourage cycling behavior and amplify the positive effects of accessibility and land use mix.

## Supplementary material

10.2196/58761 Multimedia Appendix 1 Supplementary material.

## Abbreviations

CBD: central business district
HHI: Herfindahl-Hirschman index
NBRM: negative binomial regression model
POI: point of interest
