# Supplementary material for: Moderation Effects of Streetscape Perceptions on the Associations Between Accessibility, Land Use Mix, and Bike-Sharing Use: Cross-Sectional Study
Source: JMIR Public Health Surveill. 2024 Jul 3;10:e58761. doi: 10.2196/58761 (PMC11238024; doi:10.2196/58761)
Supplement: Multimedia Appendix 1 [file publichealth-v10-e58761-s001.docx]

### Descriptive Analysis

Table S1 presents the summary statistics of bike-sharing usage, macroscale built environment elements and streetscape perceptions. The mean and standard deviation of the number of bike-sharing origins on weekends were 3,248 and 3,424, respectively, which indicates a great difference in residents’ bike-sharing usage during weekends. With regards to streetscape perceptions, there are considerable variations in lively, wealthy and safety perceptions, especially for the first two perceived factors, with the standard deviations of 5.03 and 4.20, respectively. Similar to the pattern of streetscape perceptions shown in Table 1, large differences can be found for macroscale built environment elements.

Table S1. Descriptive statistics of bike-sharing usage, macroscale built environment elements and streetscape perceptions.

| Variables | Mean | SD | Min | Median | Max |
| --- | --- | --- | --- | --- | --- |
| Bike-sharing origins ^a^ | 3.25 | 3.42 | 0.00 | 2.14 | 25.37 |
| Streetscape perceptions |  |  |  |  |  |
| Lively | 34.78 | 5.03 | 15.35 | 35.10 | 55.97 |
| Safety | 36.40 | 2.91 | 18.60 | 36.54 | 50.60 |
| Wealthy | 40.78 | 4.20 | 23.97 | 40.78 | 58.70 |
| Macroscale built environment |  |  |  |  |  |
| Population ^a^ | 2.29 | 1.94 | 0.00 | 1.96 | 18.39 |
| Average building height | 15.91 | 8.34 | 0.00 | 14.47 | 97.90 |
| Land use mix | 0.10 | 0.14 | 0.00 | 0.05 | 1.14 |
| Number of road intersections | 2.32 | 1.32 | 0.00 | 3.00 | 5.00 |
| Distance to nearest transit | 5.24 | 4.34 | 0.00 | 3.97 | 29.82 |
| Number of bus stations | 1.27 | 1.27 | 0.00 | 1.00 | 8.00 |
| Number of POIs ^a^ | 0.24 | 0.30 | 0.00 | 0.15 | 3.56 |
| Distance to the nearest park | 2.54 | 2.24 | 0.01 | 1.98 | 15.80 |
| Distance to CBD | 44.81 | 17.47 | 0.00 | 45.88 | 87.77 |

^a^  for value = original value / 1000.

**Table S2.** Effects of lively, safety and wealthy perceptions on the number of bike-sharing usage on weekends during 2018 in Shanghai, China using univariate model

| Lively ^a^ |  | Safety ^a^ |  | Wealthy ^a^ |  |
| --- | --- | --- | --- | --- | --- |
| β (SD) | *P* | β (SD) | *P* | β (SD) | *P* |
| 5.50 (0.27) | <0.001 | 5.38 (0.25) | <0.001 | 4.45 (0.23) | <0.001 |

^a^  for value = original value / 1000.


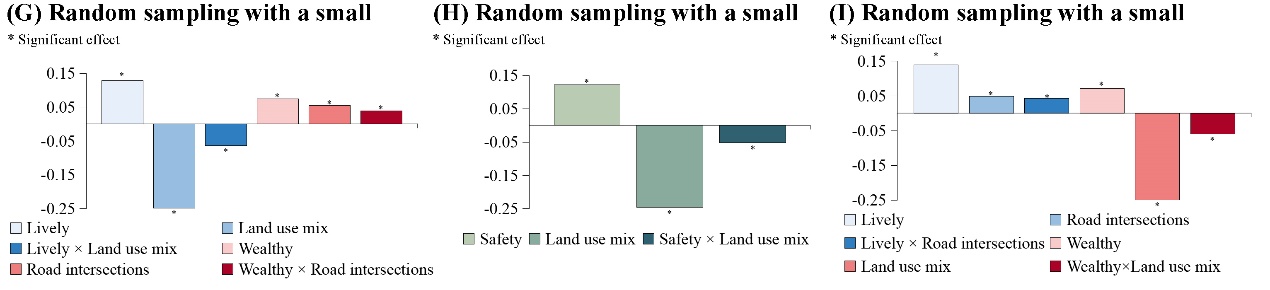


Figure S1. Sensitivity analyses of streetscape moderation effects examined at different geographic scales using a small-sample approach (G-I).
